# O9.2. IDENTIFYING PSYCHOTIC SYMPTOMS AND PREDICTING RELAPSE THROUGH SOCIAL MEDIA

**DOI:** 10.1093/schbul/sby015.246

**Published:** 2018-04-01

**Authors:** Michael Birnbaum, Asra Rizvi, Munmun De Choudhury, Sindhu Ernala, Guillermo Cecchi, John Kane

**Affiliations:** 1 Northwell Health-Early Treatment Program; 2 Zucker Hillside Hospital; 3 Georgia Institute of Technology; 4 IBM Research; 5 Northwell Health

## Abstract

**Background:**

The internet and social media provide an unprecedented opportunity to transform early psychosis intervention services. This study aimed to capture concerning patterns of social media activity associated with the onset and persistence of psychotic symptoms.

**Methods:**

Facebook and Twitter archives were extracted from over 150 participants with psychotic disorders, mood disorders and healthy controls. Machine learning was used to build classifiers aiming to identify patterns and distinguish between groups.

**Results:**

Linguistic analysis of Twitter commentary identified significantly increased use of interpersonal pronouns (p < 0.001), decreased emphasis on friendship (p < 0.001) and increased emphasis on health (p < 0.001) in individuals with psychosis. Preliminary classifiers correctly recognized participants with psychotic disorders (n=62) from healthy controls (n=24) with an average accuracy of 80% and distinguished participants with psychosis from those with mood disorders (n=39) with an average accuracy of 70%. Further analysis identified shifts in language use of participants with psychosis who experience a relapse (n=18) including significant increases in the use of swearing (p<0.05), first-person pronouns (p<0.05) and negations (p<0.05). We additionally identified significant differences in the profile pictures (p<0.005) and structure of messages posted (p<0.005) by youth with psychosis who experienced a psychotic relapse.

**Discussion:**

Identifying markers in social media activity associated with worsening psychotic symptoms offers the prospect that social media may be a clinically useful tool to identify patients in the earliest phases of relapse.

